# Molecular Characterization of Hepatitis B Virus Infection in a Patient with Cutaneous Lupus Erythematosus

**DOI:** 10.3390/diagnostics12112866

**Published:** 2022-11-19

**Authors:** Umbertina Villano, Elida Mataj, Maria Dorrucci, Francesca Farchi, Carmelo Pirone, Catia Valdarchi, Michele Equestre, Elisabetta Madonna, Roberto Bruni, Giulio Pisani, Antonio Martina, Matteo Simeoni, Giancarlo Iaiani, Massimo Ciccozzi, Anna Rita Ciccaglione, Fabrizio Conti, Fulvia Ceccarelli, Alessandra Lo Presti

**Affiliations:** 1 Department of Infectious Diseases, Istituto Superiore di Sanità, 00161 Rome, Italy; 2 Instituti i Shendetit Publik (ISHP), Alessander Moisiu No. 80, Tirane, Albania; 3 Lupus Clinic, Rheumatology, Dipartimento di Scienze Cliniche, Internistiche, Anestesiologiche e Cardiovascolari, Sapienza Università, 00185 Rome, Italy; 4 Department of Cell Biology and Neuroscience, Istituto Superiore di Sanità, 00161 Rome, Italy; 5 Center for Immunobiological Research and Evaluation, National Institute of Health, 00161 Rome, Italy; 6 Department of Tropical and Infectious Diseases, Aou Policlinico Umberto I, 00185 Rome, Italy; 7 Unit of Medical Statistics and Molecular Epidemiology, University Campus Bio-Medico of Rome, 00128 Rome, Italy

**Keywords:** Hepatitis B virus, Lupus erythematosus, autoantibodies, phylogenetic analysis, mutations

## Abstract

Hepatitis B virus (HBV) infection is a serious global health problem. Patients with autoimmune diseases, such as Lupus Erythematosus, are exposed to a higher risk of acquiring infections. In this study, a molecular characterization, genomic investigation of the Hepatitis B virus, polymerase (P) and surface (S) genes, from a patient affected by Cutaneous Lupus Erythematosus (CLE), was presented. Viral DNA was extracted from 200 μL of serum, and the HBV-DNA was amplified by real-time polymerase chain reaction (PCR) with the Platinum Taq DNA Polymerase. The PCR products were purified and sequencing reactions were performed. A phylogenetic analysis was performed through maximum likelihood and Bayesian approaches. The HBV CLE isolate was classified as sub-genotype D3 and related to other Italian HBV D3 genomes, and some from foreign countries. No drug resistant mutations were identified. One mutation (a.a. 168 M) was located in the last part of the major hydrophilic region (MHR) of the surface antigen (HBsAg). Moreover, three sites (351G, 526Y, 578C) in the polymerase were exclusively present in the CLE patient. The mutations identified exclusively in the HBsAg of our CLE patient may have been selected because of the Lupus autoantibodies, which are characteristic in the Lupus autoimmune disease, using a possible molecular mimicry mechanism.

## 1. Introduction

Hepatitis B virus infection is a serious global health problem [1]. Over 350 million individuals chronically infected with HBV are at high risk of developing liver cirrhosis and hepatocellular carcinoma (HCC) [2]. Based on its genetic divergence, HBV has been classified into 10 genotypes [3], which also includes the newest genotype J identified in Japan [4]. The majority of the HBV genotypes are further divided into sub-genotypes with a specific geographic distribution, as previously described [5,6]. The prevalent genotypes in Europe are A (mainly subgenotype A2) and D (mainly subgenotypes D1, D2, D3) and in Italy D3 has been reported as the prevalent sub-genotype [6].

Compared to healthy individuals, patients with autoimmune diseases such as Lupus Erythematosus are exposed to a higher risk of acquiring infections. Systemic lupus erythematosus (SLE) is a multisystem autoimmune disorder predominantly affecting women of child-bearing age, with a chronic relapsing–remitting course. Moreover, an exclusively cutaneous condition has been also widely described, the so-called Cutaneous Lupus Erythematosus (CLE) [7,8]. The clinical heterogeneity of CLE is well recognized. Four major subtypes are defined according to the modified Gilliam grouping system [9]; among them, the discoid LE (DLE) is the most common form of chronic CLE. In a previous study, we evaluated the prevalence of HBV and HCV infection, together with the possible associations with clinical, epidemiological characteristics in a large cohort including patients affected by SLE and CLE attending the Lupus Clinic of the Rheumatology Unit, Sapienza University of Rome (Italy) [10]. The viral infections have been implicated in autoimmune diseases’ pathogenesis [11]. In this study, we performed the molecular characterization and genomic diversity investigation of the Hepatitis B virus in order to determine the phylogenetic relationships and mutations on the HBV polymerase (P) and surface (S) genes combined to clinical description from a CLE patient belonging to our cohort.

## 2. Case Presentation

Here, we report a 63 years old female Italian patient, born in Calabria and living in Rome (Lazio Region). She was diagnosed with Discoid Lupus Erythematosus (DLE) in 2009 at the Lupus Clinic of the Rheumatology Unit (Sapienza University of Rome, Italy). At the time of diagnosis, we found a mild positivity for anti-nuclear antibodies (ANA, IFI on Hep2, 1:80, homogenous pattern), that disappeared after 2 years. Starting from 2010, she started treatment with hydroxychloroquine 400 mg/die, with fast improvement of skin lesions. The patient denies having traveled abroad; moreover, the patient has never received blood transfusions, tattoos or participated in drug use. The patient, from 1985 to 1996, received three gastroscopies due to epigastralgia. She receives regular dental care. In 1998, she underwent a basal cell carcinoma removal surgery. The patient was not vaccinated for HBV.

In the family history, the patient reported that her father had a HBV infection at 60 years, but no other data were available. This study was approved by the local Ethical Committee (Prot. PRE-16/18, 15 January 2018—Istituto Superiore di Sanità), and the patient provided written informed consent [10]. HBV serological markers, DNA extraction and HBV-DNA levels quantification were previously reported [10]. In particular, HBV-DNA levels in the plasma were detected and quantified by the COBAS AMPLIPREP/COBAS TAQMAN HBV TEST, V2.0 with a claimed lower limit of detection of 20 IU/mL and a claimed upper limit of quantification of 1.7 × 10^8^ IU/mL. HBV-DNA was extracted from 200 μL of the serum sample at the National Institute of Health (Istituto Superiore di Sanità) (Rome, Italy), using the EZ1 Virus Mini Kit v.2.0 (Qiagen, Hilden, Germany), and following the manufacturer’s instructions. HBV-DNA was amplified by real-time polymerase chain reaction (PCR) with the Platinum Taq DNA Polymerase (Invitrogen by Life Technologies Corporation). The PCR products were purified using the QIAquick PCR Purification Kit (Qiagen Hilden, Germany) in accordance with the manufacturer’s instructions. Sequencing reactions were performed using the GenomeLab DTCS Quick Start KiT (Beckman Coulter, Inc., Fullerton, CA, USA) and were run on an automated DNA sequencer (Beckman Coulter, Inc., Fullerton, CA, USA). Raw output sequences were analyzed by the Chromas software (http://www.technelysium.com.au/chromas.html accessed on 24 June 2020) and the BioEdit Package [12]. The analysis of the serology demonstrated this patient to be positive for the Hepatitis B surface antigen (HBsAg); thus, in 2009, HBV infection was confirmed. The anti-HBc titre was 0.01. The AST and ALT values were 17 and 22 U/L, respectively. The serum blood hepatitis B virus deoxyribonucleic acid (HBV DNA) concentration was 133.0 copies/mL. Based on the above reported clinical parameters and previous history, the patient was classified as a chronic hepatitis B. Moreover, the subject was confirmed negative for an HCV infection [10].

Six different datasets were built. The first one contained the HBV polymerase gene sequence from the CLE patient (named 104, Accession Number: OP572234), plus 40 genotype/sub-genotype specific reference HBV polymerase gene sequences downloaded from the National Centre for Biotechnology Information (NCBI) (http://www.ncbi.nlm.nih.gov/ accessed on 14 July 2020); this dataset has been used to establish the sub-genotype of our isolate. The second dataset included the HBV S gene sequence from the CLE patient (named 104) plus 40 genotype/sub-genotype specific reference HBV S gene sequences downloaded from the National Centre for Biotechnology Information (NCBI) (http://www.ncbi.nlm.nih.gov/ accessed on 14 July 2020); this dataset has been used to confirm the sub-genotype of our isolate. The third dataset included the HBV polymerase gene sequences from the CLE patient (named 104, accession Number: OP572234) plus 93 HBV D3 genotype polymerase sequences collected from different countries in order to evaluate the phylogenetic relationships and intermixing, with respect to foreign sequences. The fourth dataset was also composed by the HBV S gene sequences from the CLE patient (named 104, accession Number: OP572234) plus 93 HBV D3 genotype S sequences collected from different countries, to evaluate the phylogenetic relationships and intermixing, with respect to foreign sequences. The fifth and sixth dataset contained a total of 124 sequences: including our isolate sequence, together with 123 D3 genotype sequences downloaded from the National Centre for Biotechnology Information (NCBI) (http://www.ncbi.nlm.nih.gov/ accessed on 10 March 2022), and were used respectively for HBsAg and polymerase alignments, in order to investigate the exclusive presence of mutations in our isolate, with respect to a high number of other D3 genotype sequences available from databases. The sequences of all datasets were aligned by using Bioedit software, followed by manual editing [12]. The maximum likelihood phylogenetic tree together with the best fitting evolutionary models for the first and second dataset were estimated through IQTREE [13]. The statistical support for the internal branches of the ML tree was evaluated by bootstrapping (1000 replicates) and fast likelihood-based sh-like probability (SH-aLRT). The alignments of the third and fourth dataset were also analyzed through Mr Bayes [14,15]. A Markov chain Monte Carlo search was conducted for 10 × 10^6^ generations using tree sampling every 100th generation, with the GTR + I + G substitution model and a burn-in fraction of 25%. Statistical support for specific clades and clusters was obtained by calculating the posterior probability of each monophyletic clade (posterior probability > 0.90), and a posterior consensus tree was generated after a 25% burn-in. Mutations predictions were performed in the polymerase and S gene (HBsAg) of the HBV isolate number 104 through Geno2pheno HBV 2.0 (https://hbv.geno2pheno.org/ accessed on 11 August 2020) together with the prediction of resistance of the virus to five antiviral drugs (Lamivudine, Adefovir, Entecavir, Tenofovir and Telbivudine). In addition, the alignments of the fifth (HBsAg) and sixth (polymerase) dataset were subjected to visual inspection through Bioedit [12], in order to confirm the mutations resulting exclusively in our isolate.

**Figure 1:**
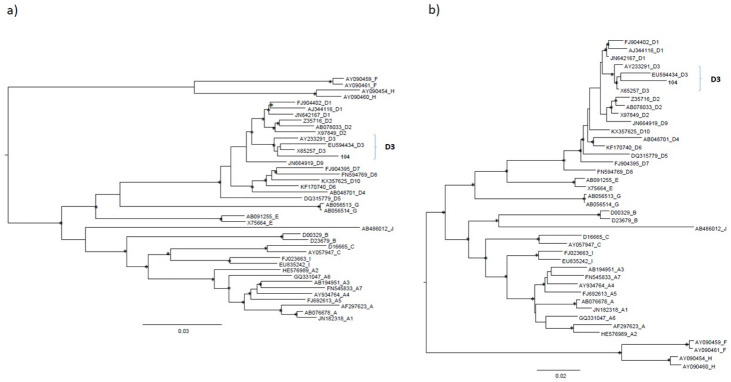
The maximum likelihood phylogenetic tree built on the first (**a**) and second dataset of HBV (**b**). The trees were rooted using the midpoint rooting method. The scale bar at the bottom of the tree represents 0.03 and 0.02 nucleotide substitutions per site respectively for the first and second dataset. An asterisk along the branches represents a SH-aLRT ≥ 80% and UFboot ≥ 95%. Accession numbers of the sequences are indicated in the first part of the tip names followed by HBV genotype/sub-genotype. The accession number for isolate 104 is: OP572234. The HBV sub-genotype D3 cluster was highlighted by brackets.

The maximum likelihood phylogenetic tree built on the first and second dataset demonstrated that the sequence of the isolate 104 (CLE patient) belonged to sub-genotype D3 and was located in a statistically supported cluster (Figure 1a and Figure 1b, respectively).

**Figure 2:**
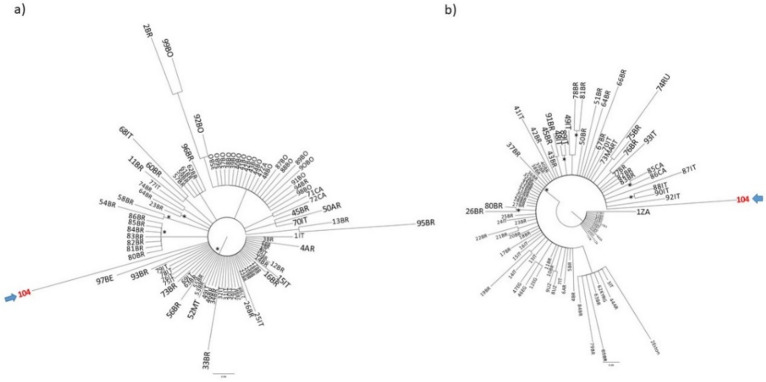
Bayesian phylogenetic analysis on the third (**a**) and fourth dataset of HBV (**b**). Branch lengths were estimated with the best fitting nucleotide substitution model according to a hierarchical likelihood ratio test and were drawn to scale with the bar at the bottom indicating 0.08 (**a**,**b**) nucleotide substitutions per site. The trees were rooted using the midpoint rooting method. One * along the branches represent significant statistical support for the clade subtending that branch (posterior probability > 90%). The accession number for isolate 104 is OP572234. The accession numbers of the sequences of the third and fourth dataset are reported in Table S1.

The Bayesian phylogenetic tree performed on the polymerase gene D3 sequences (Figure 2a) indicated that isolate 104 was related to other Italian D3 isolates. Sequences from Brazil, Argentina, Martinique and Belgium appeared also proximal to our isolate. The tree exhibited on panel b indicates that the isolate 104 belonged to a major clade (without forming statistically supported internal clusters). It appears to be intermixed mainly with other sequences from Italy, Brazil, Martinique and Canada. Some sequences from Uzbekistan, Egypt, Argentina and Kyrgyzstan were also identified in this major clade.

Geno2Pheno confirmed the assignment of our isolate 104 to sub-genotype D3 (similarity to sub-genotype profile = 97.99%). The mutations identified through Geno2Pheno for the polymerase and HBsAg in isolate 104 reported in Table 1a) and were confirmed through visual inspection of the alignments. Six AA residues in the polymerase (five of them belonging to the RT domain) were found mutated in the isolate Id. 104, with respect to the reference D3 (Acc. Number X65257). In particular, three of the above reported variations (351G, 526Y, 578C) appeared specific and present only in our isolate if compared to the 93 HBV D3 reference sequences included in this dataset.

Seven AA residues in the HBsAg isolate 104 sequence were found mutated with respect to the reference D3 (Acc. Number X65257), six of them identified through Geno2Pheno and one confirmed through the alignment. In particular, five of them (168M, 268S, 344I, 394S, 396V) appeared to be specific and present only in our isolate if compared to the 93 HBV D3 reference sequences included in this dataset. By analyzing a larger number of HBV D3 sequences (*n* = 123, fifth and sixth dataset for HBsAg and polymerase protein, respectively, whose accession numbers are indicated in Table S1) we found that, five residues were confirmed exclusive in our isolate 104 HBsAg protein sequence and reported in Table 1b. Moreover, three sites (351G, 526Y, 578C) were confirmed in the polymerase protein as exclusive to the CLE patient. The resistance analysis through Geno2Pheno revealed no drug resistance; accordingly, the prediction of susceptibility to Lamivudine, Adefovir, Entecavir, Tenofovir and Telbivudine.

## 3. Discussion

Systemic Lupus Erythematosus (SLE), a chronic autoimmune disease, is characterized by a multifactorial etiology, in which genetic and environmental factors interplay [16,17]. The disease is characterized by chronic inflammation, production of different autoantibodies, complement activation and immune-complex deposition, resulting also in tissue damage [16]. The viral infections can have a role in disease development and exacerbation [16,18,19]. This study focused on a form that was exclusively cutaneous, and defined the so-called Cutaneous Lupus Erythematosus (CLE). HBV infections have posed a major public-health problem worldwide, representing the major cause leading to chronic liver disease, but few data are available in literature concerning these infections in Lupus Erythematosus patients. This study reported for the first time, to the best of our knowledge, the clinical description and phylogenetic characterization of HBV sequences derived from a patient affected by Cutaneous Lupus Erythematosus in Italy. HBV genotypes and sub-genotypes have distinct geographical and ethnic distribution [20]. In particular, the HBV genotype D has a relatively broad geographical distribution, being found in regions including the Mediterranean, North-eastern Europe, India, Oceania and parts of southern Africa [21]. It is characterized by a high degree of heterogeneity. Here, we demonstrated the circulation of genotype D, sub-genotype D3 in our patient, in agreement with the findings that HBV/D3 is highly prevalent in Italy [22,23]. Using phylogenetic analysis, several studies conducted in different parts of the world have demonstrated that HBV migrates with its hosts. Our data demonstrated that the HBV CLE isolate sequences were related to other Italian D3 genomes, but also with other foreign genomes (i.e., Brazil, Argentina, Martinique, Canada and Belgium, which are also proximal to our isolate). The relationship of HBV/D3 isolated in Brazil with sequences from Italy was previously observed by other authors [24] and explained by an intense European immigration to Brazil, mostly due to Italian people occurring in the XIX and XX centuries. The reverse transcriptase of HBV polymerase consists of 344 amino acids, starting with the highly conserved EDWGPCDEHG motif and partially overlapping with the HBV surface antigens. Mutations within reverse transcriptase probably affect the replication capacity of HBV, which in turn might alter the antigenicity, encapsidation and virulence of the virus as well as the generation of drug resistance [25,26,27]. In regard to the HBV polymerase mutations identified in this study, no drug resistant mutations in the reverse transcriptase were identified.

The determination of the resistance profile is crucial in choosing the right antiviral agent to initiate therapy, to monitor, to optimize the best treatment and, consequently, reduce the progression of the disease. The prediction of susceptibility to Lamivudine, Adefovir, Entecavir, Tenofovir and Telbivudine was here identified.

The HBV surface antigen (HBsAg) includes the main epitopes recognized by neutralizing antibodies. The central core of HBsAg, comprising amino acids 99–169, which is referred to as the major hydrophilic region (MHR), is exposed on the surface and is involved in binding to antibodies directed against HBsAg and comprises the “a determinant” (aa 124–147). Changes inside the “a determinant” can lead to conformational changes and can affect the binding of neutralizing antibodies.

The mutations identified in our study in the HBsAg protein did not fall within those highlighted by Lazarevic I. et al. [28] as clinically relevant with a possible related role in vaccine escape and OBI; in only one of them the a.a. 168 (mutation 168 M identified in our isolate) was in any way located in the last part of the MHR. Mutations in certain regions of the HBV genome could be responsible for an unwanted clinical outcome or the evasion of detection by diagnostic tools, thus making the monitoring for these mutations a necessity in the proper evaluation of patients. A possible explanation for the mutations identified exclusively in the HBsAg protein of our CLE patient could be that they can be selected as a consequence of the Lupus auto-antibodies, characteristic in Lupus autoimmune disease, using a possible mechanism of molecular mimicry [29,30]. Patients with SLE demonstrate enhanced expression of miR-30e, pro-inflammatory cytokines, type-I interferons, or type-I interferon-inducible genes. Some authors reported that several negative regulators of innate immunity play a crucial role in the development of autoimmune diseases. In SLE pathogenesis, the enhanced expression of miR-30e might play a crucial role by suppressing the expression of negative regulators of the innate immune signaling pathway, which in turn enhances innate immune cytokines and contributes to the development or severity of the disease [30]. Before drawing conclusions, a limit of this study consists certainly in being a case report, based on only one patient affected by CLE with chronic HBV infection, available from our cohort. Future multi-center studies should be applied in order to confirm this assumption.

## 4. Conclusions

Patients with autoimmune diseases such as Lupus Erythematosus are exposed to a higher risk of acquiring infections. It is important to monitor by molecular characterization the HBV mutations potentially selected in these fragile populations.

## Figures and Tables

**Table 1:** (**a**). Mutations identified through Geno2Pheno for the polymerase and HBsAg. For polymerase, the amino acid positions are referred with respect to the complete polymerase (Acc Number AB778116) starting from the first methionine. For HBsAg, the amino acid positions are referred with respect to HBsAg complete (Acc Number AY040803) starting from the first methionine. The symbols (^#^, °, ^§^) indicate changes in RT, resulting also in changes in HBsAg. (**b**) Mutations identified exclusively in our isolate, with respect to the 123 D3 genotype sequences of the fifth and sixth dataset.

	Isolate 104	Amino Acid in Reference Acc Number: X65257
**(a)**		
polymerase	339 H	Y
	351G ^#^	W
	402H	Y
	468G	R
	526Y °	F
	578C ^§^	S
HBsAg	168M ^#^	I
	266 T	I
	268 S	L
	344 I °	F
	381 N	S
	394 S ^§^	F
	396 V	L
**(b)**		
polymerase	351G	W
	526Y	F
	578C	S
	168M	I
	268 S	L
HBsAg	344 I	F
	394 S	F
	396 V	L

## Data Availability

The sequence has been submitted to GenBank—NCBI and the accession number is: OP572234.

## Supplementary Materials

The following supporting information can be downloaded at: https://www.mdpi.com/article/10.3390/diagnostics12112866/s1, Table S1: The accession numbers of the HBV sequences used in molecular investigations for the third, fourth, fifth and sixth dataset.
