# Supplementary material for: Molecular Characterization of Hepatitis B Virus Infection in a Patient with Cutaneous Lupus Erythematosus
Source: Diagnostics (Basel). 2022 Nov 19;12(11):2866. doi: 10.3390/diagnostics12112866 (PMC9689093; doi:10.3390/diagnostics12112866)
Supplement: Supplementary file 1 [file diagnostics-12-02866-s001.zip › diagnostics-1877925-supplementary.pdf]

Table S1. The Accession numbers of the HBV sequences used in molecular investigations for the third, fourth, fifth and sixth dataset.

Accession numbers	Tip names
**third dataset**	
EU908801.1	1IT
MH724235.1	2BR
KY809965.1	3BR
JN688708.2	4AR
MN310710.1	6BR
MN310709.1	7BR
MN702735.1	8IT
MN702714.1	9IT
MH724214.1	10BR
KY810010.1	11BR
KY809974.1	12BR
KY809961.1	13BR
KP090181.1	14BR
KP091719.1	15IT
JF815660.1	16BR
JF815651.1	17BR
JF815645.1	18BR
JF815634.1	19BR
JF815633.1	20BR
JF815622.1	21BR
JF815610.1	22BR
JF815609.1	23BR
EU908826.1	25IT
KY809976.1	26BR
MN310715.1	27BR
MN310708.1	28BR
MN702750.1	29IT
MN702732.1	30IT
MN702726.1	31IT
MN702711.1	32IT
MH724243.1	33BR
MH724233.1	34BR
MH464853.1	35BO
MH464852.1	36BO
MH464850.1	37BO
MH464847.1	38BO
MH464846.1	39BO
MH464845.1	40BO
MH464839.1	41BO
MH464837.1	42BO
MH464832.1	43BO

MH464831.1	44BO
KY810011.1	45BR
KY809979.1	46BR
KT347090.1	47ZA
KR139747.1	48BO
KP091720.1	49IT
JN688689.2	50AR
HE974377.1	52MT
JF815656.1	53BR
JF815655.1	54BR
JF815637.1	55BR
JF815635.1	56BR
JF815632.1	57BR
JF815627.1	58BR
JF815626.1	59BR
JF815625.1	60BR
JF815624.1	61BR
JF815616.1	62BR
JF815615.1	63BR
JF815608.1	64BR
EU908830.1	68IT
EU908809.1	69IT
X65257.1	70IT
GQ922002.1	71CA
GQ922001.1	72CA
MH724234.1	73BR
MN310713.1	74BR
MN702749.1	75IT
MN702744.1	76IT
MN702729.1	77IT
MN702724.1	78IT
MN702723.1	79IT
MH724248.1	80BR
MH724230.1	81BR
MH724229.1	82BR
MH724228.1	83BR
MH724227.1	84BR
MH724225.1	85BR
MH724224.1	86BR
MH464854.1	87BO
MH464848.1	88BO
MH464843.1	89BO
MH464836.1	90BO
MH464834.1	91BO
MF979162.1	92BO
KY810013.1	93BR

KY809981.1	94BR
KY809959.1	95BR
KY809957.1	96BR
KX372199.1	97BE
MH464841.1	98BO
MH464838.1	99BO
**fourth dataset**	
AY233291	1ZA
EU594434	2Eston
X65257	3IT
MH724235.1	4BR
KY809965.1	5BR
JN688708	6AR
EU908801	7IT
KY210496.1	8UZ
KY210495	9UZ
MN310710	10BR
MN310709	11BR
MT004769	12EG
MN702750.1	13IT
MN702735.1	14IT
MN702732.1	15IT
MN702714	16IT
MH724234	17BR
MH724214	18BR
KY810010	19BR
KY809979.1	20BR
KY809974.1	21BR
KY809961.1	22BR
KP090181.1	23BR
KP091719.1	24IT
JF815660.1	25BR
JF815655.1	26BR
JF815651.1	27BR
JF815645.1	28BR
JF815634.1	29BR
JF815633.1	30BR
JF815632.1	31BR
JF815626.1	32BR
JF815624.1	33BR
JF815622.1	34BR
JF815619.1	35BR
JF815616.1	36BR
JF815612.1	37BR
JF815611.1	38BR
JF815610.1	39BR

JF815609.1	40BR
EU908826.1	41IT
KY809976.1	42BR
MN310715.1	43BR
JN688689.2	44AR
MN310708.1	45BR
MT004773.1	46EG
MT004770.1	47EG
MN702724.1	48IT
MN702711.1	49IT
MH724243.1	50BR
MH724233.1	51BR
MH464853.1	52BO
MH464852.1	53BO
MH464850.1	54BO
MH464847.1	55BO
MH464846.1	56BO
MH464845.1	57BO
MH464839.1 H	58BO
MH464837.1	59BO
MH464832.1	60BO
MH464831.1	61BO
KY210523.1	62KYRG
KY810011.1	63BR
KY810003.1	64BR
KY809985.1	65BR
KY809959.1	66BR
KY809957.1	67BR
KT347090.1	68ZA
KR139747.1	69BO
KP091720.1	70IT
KM519455.1	71ZA
KC752145.1	72IN
HE974377.1	73MART
JX090694.1	74RU
JF815676.1	75BR
JF815656.1	76BR
JF815637.1	77BR
JF815635.1	78BR
JF815628.1	79BR
JF815627.1	80BR
JF815625.1	81BR
JF815615.1	82BR
JF815608.1	83BR
HM101109.1	84BR
GQ922002.1	85CA

GQ922001.1	86CA
EU908830.1	87IT
EU908823.1	88IT
EU908811.1	89IT
EU908809.1	90IT
MN310713.1	91BR
MN702749.1	92IT
MN702744.1	93IT
**fifth and sixth dataset**	
AB583679.1	
AB674437.1	
DQ315776.1	
DQ315777.1	
EU594382.1	
EU594434.1	
EU594435.1	
EU594436.1	
FJ692506.2	
FJ692507.2	
GQ922000.1	
GQ922001.1	
GQ922002.1	
HE815465.1	
HE974377.1	
HE974379.1	
HQ236014.1	
HQ236015.1	
HQ236016.1	
JF754625.1	
JN688678.2	
JN688679.2	
JN688683.2	
JN688685.2	
JN688689.2	
JN688708.2	
JN688710.2	
JN688711.2	
JN688712.2	
JN688713.2	
JN688715.2	
JN688716.2	
JN688722.2	
JQ023666.1	
JX470760.1	
JX898686.1	
JX898687.1	

JX898688.1	
JX898689.1	
JX898690.1	
JX898691.1	
JX898692.1	
JX898693.1	
JX898694.1	
JX898695.1	
JX898696.1	
JX898697.1	
JX898698.1	
JX898699.1	
KC012652.1	
KF679988.1	
KF679989.1	
KF679990.1	
KF679991.1	
KF679992.1	
KF679993.1	
KJ647349.1	
KJ647351.1	
KJ647355.1	
KM359442.1	
KM386676.1	
KM519455.1	
KM577663.1	
KM577664.1	
KM577665.1	
KM577666.1	
KM577667.1	
KM606745.1	
KP090177.1	
KP090178.1	
KP090179.1	
KP090180.1	
KP090181.1	
KP322602.1	
KT749845.1	
KU736926.1	
KU736927.1	
MG877709.1	
MG877711.1	
MG877718.1	
MG877719.1	
MG877720.1	
MG877726.1	

MG877727.1	
MG877728.1	
MH724214.1	
MH724215.1	
MH724218.1	
MH724219.1	
MH724220.1	
MH724221.1	
MH724222.1	
MH724224.1	
MH724225.1	
MH724226.1	
MH724227.1	
MH724228.1	
MH724229.1	
MH724230.1	
MH724232.1	
MH724233.1	
MH724234.1	
MH724235.1	
MH724237.1	
MH724239.1	
MH724240.1	
MH724242.1	
MH724243.1	
MH724245.1	
MH724248.1	
MH724249.1	
MH724250.1	
MK507912.1	
MK541688.1	
MN310708.1	
MN310709.1	
MN310710.1	
MN310711.1	
MN310712.1	
MN310713.1	
MN310714.1	
MN310715.1	
MT210033.1	
